# In Vitro Propagation of Variegated *Cymbidium lancifolium* Hooker

**DOI:** 10.3390/plants14162551

**Published:** 2025-08-16

**Authors:** Iro Kang, Iyyakkannu Sivanesan

**Affiliations:** 1 Department of Horticulture, College of Agriculture & Natural Resources, Michigan State University, East Lansing, MI 48824, USA; kanghye4@msu.edu; 2 Department of Environmental Health Science, Institute of Natural Science and Agriculture, Konkuk University, 1 Hwayang-dong, Gwangjin-gu, Seoul 05029, Republic of Korea

**Keywords:** asymbiotic seed germination, auxin, cytokinin, GA_3_, coconut water, orchids

## Abstract

Variegated *Cymbidium lancifolium* is a highly valued ornamental plant sought after in local and international markets. The commercial production of variegated *C. lancifolium* through traditional propagation methods faces significant challenges, such as low propagation rates and prolonged growth periods. This study aims to develop effective in vitro propagation techniques for variegated *C. lancifolium* through asymbiotic seed germination to enhance production efficiency and meet market demand. We examined the effects of various plant growth regulators and coconut water (CW) on in vitro seed germination. The highest germination percentage (46.8%) was recorded in Murashige and Skoog (MS) medium supplemented with 50 mL/L CW, 4.0 µM α-naphthalene acetic acid (NAA), 2.3 µM kinetin (KN), and 2.9 µM gibberellic acid (GA_3_). Seed-derived rhizomes were placed on MS medium containing indole-3-acetic acid (IAA), indole-3-butyric acid (IBA), and NAA for proliferation. Among the auxins, NAA was the most effective, significantly increasing rhizome proliferation, with the highest number (17.4) and length (2.1 cm) observed at 5.0 µM. The rhizome explants were cultured in MS medium enriched with kinetin (KN), N^6^-(2-isopentenyl)adenine (2-IP), and N^6^-benzyladenine (BA) to promote plantlet regeneration. Of the cytokinins tested, BA at 10.0 µM resulted in the highest rate of plantlet regeneration (79.4%), the greatest number of plantlets (4.4 per culture), and notable plantlet height (8.5 cm). We obtained plantlets with dark green leaves, light green leaves, and distinct variegation patterns. They were transferred to three different substrate mixtures for acclimatization. The substrate made of orchid stone (30%), wood bark (30%), coconut husk chips (20%), and perlite (20%) supported the highest survival rate (95.9%). This study successfully established optimized in vitro propagation techniques for variegated *C. lancifolium*, enabling enhanced germination, rhizome proliferation, and plantlet regeneration to meet the growing market demand.

## 1. Introduction

Orchids are among the most advanced monocotyledonous plants and have been known to humans for a considerable time. The genus *Cymbidium* Swartz, belonging to the Orchidaceae family, comprises 70 species [1] and is often referred to as the orchids of the East. These species are found in temperate regions such as Korea, Japan, and China and have been valued as noble plants since ancient times [1,2]. *Cymbidium* species are prized ornamental plants that are frequently sold as cut flowers and as potted plants. They are also used in landscape gardening and can be incorporated into hanging baskets [3,4]. *Cymbidium lancifolium*, a small terrestrial herb, sometimes grows saxicolous or epiphytic at elevations ranging from 300 to 2200 m. It is native to Korea and the tropical and subtropical regions of Asia [1]. *C. lancifolium* is recognized as a critically endangered species and is listed in the Korean Red Data Book [5]. Therefore, conservation efforts for *C. lancifolium* are needed.

The conventional propagation of *C. lancifolium* through both asexual and sexual methods is relatively slow due to its slow growth and the challenges associated with seed germination under natural conditions [5]. Therefore, effective techniques for the large-scale propagation of this species for commercial use are essential. Tissue culture methods have been widely used to propagate various species of orchids. Among these in vitro propagation techniques, asymbiotic seed germination is often employed to produce rare and commercially important orchids, including *Cymbidium* species [6]. This technique is also useful for introducing new isolates, genotypes, and cultivars in a short period. Numerous reports detail the in vitro propagation of *Cymbidium* species via asymbiotic seed germination; however, this method is significantly influenced by several factors, including genotype, seed age, type and composition of the culture medium, plant growth regulators (PGRs), organic additives, and culture environment [6,7,8,9].

Li et al. [10] reported that seeds of *Cymbidium faberi* ‘Jiepeimei’ × *Cymbidium sinense* ‘Qijianheimo’ collected 90 and 105 days after pollination failed to germinate in vitro. However, seeds collected 120, 135, and 150 days after pollination successfully germinated on Knudson medium. Hossain et al. [11] noted that seeds of *Cymbidium giganteum* achieved an impressive 100% germination rate in vitro when cultured on Phytomax or Mitra media supplemented with either peptone (2 g/L) or 6-benzylaminopurine (BAP, 1 mg/L). Similarly, *Cymbidium elegans* seeds germinated best on MS medium with 1 mg/L BAP [12]. In contrast, *Cymbidium iridioides* seeds germinated optimally in MS medium with 1 mg/L each of BAP and α-naphthalene acetic acid (NAA) [13]. Seeds obtained from a nine-month-old seed capsule of *Cymbidium aloifolium* exhibited the highest seed germination rate of 49% on Murashige and Skoog (MS) medium, irrespective of the addition of 0.5 mg/L BAP [14]. In contrast, seeds from seven-month-old *Cymbidium aloifolium* seed capsules germinated best (98.3%) on MS medium enriched with 0.5 mg/L each of BAP and NAA [15]. Furthermore, the highest in vitro seed germination for *Cymbidium findlaysonianum* was achieved by culturing seeds on Vacin and Went medium enriched with a combination of activated charcoal (AC, 0.2%), banana homogenate (5%), coconut water (CW, 15%), potato homogenate (5%), and 20 g/L sucrose [16]. In another study, *Cymbidium nanutum* immature seeds germinated best (45%) on half-strength MS medium containing 0.5 mg/L NAA, 30 g/L sucrose, AC (1 g/L), and coconut milk (100 mL/L) [17].

**Figure 1:**
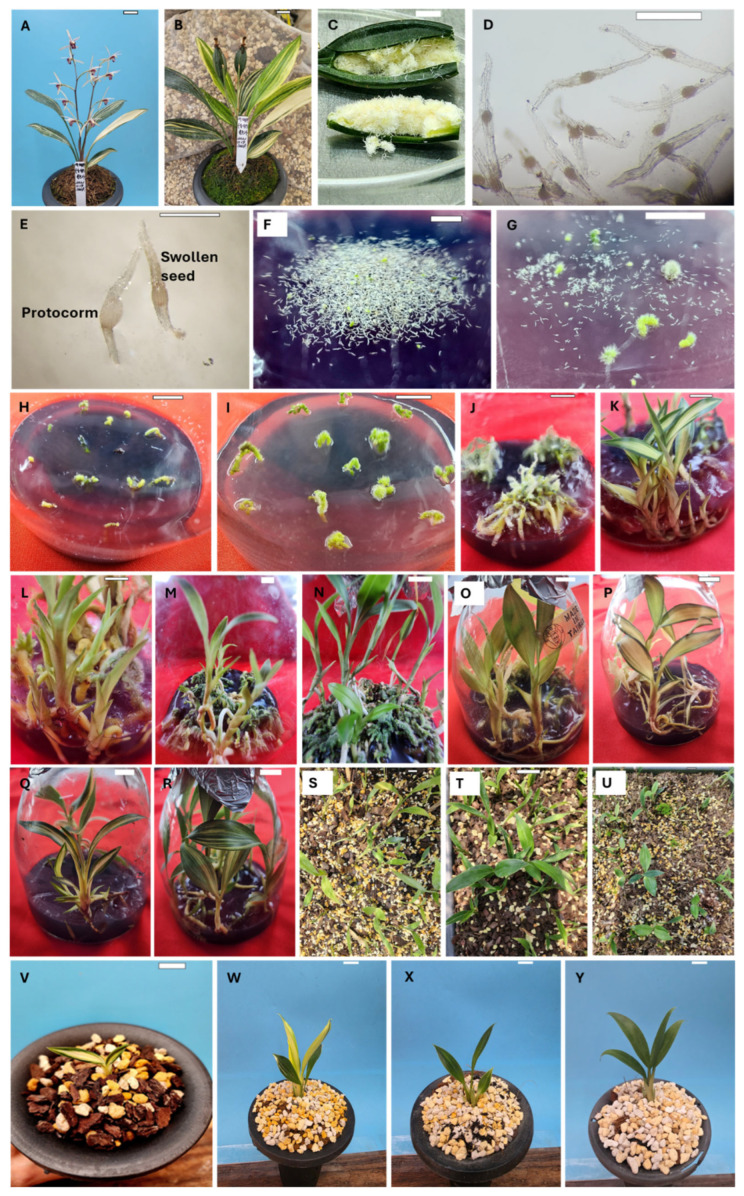
In vitro propagation of variegated *C. lancifolium*. (**A**) self-pollinated flowering plant of variegated *C. lancifolium*; (**B**) self-pollinated plant of variegated *C. lancifolium* developing mature seed capsules; (**C**) longitudinally cut seed capsule showing numerous seeds; (**D**) close-up view of mature seeds containing embryos; (**E**) formation of swollen seeds and protocorms; (**F**) seeds developing white and green protocorms; (**G**) formation of rhizomes from the protocorms; (**H**–**J**) various stages of rhizome growth and proliferation; (**K**) plantlets regenerated from the rhizome displaying prominent marginal and interveinal variegation; (**L**) regeneration of light green plantlets; (**M**) regeneration of yellow-green plantlets; (**N**) regeneration of dark green plantlets; (**O**) well-developed light green plantlets; (**P**) plantlets exhibiting central striped variegation; (**Q**) plantlets showing a combination of central and marginal variegation; (**R**) plantlets displaying sectorial and mild marginal variegation; (**S**) plantlets acclimatized in medium consisting of orchid stone (30%), wood bark (30%), coconut husk chip (20%), and perlite (20%); (**T**) plantlets acclimatized in medium consisting of orchid stone (50%) and wood bark (50%); (**U**) plantlets acclimatized in medium consisting of orchid stone (50%), coconut husk chip (10%), coconut fiber (20%), and perlite (20%); (**V**) acclimatized plantlet transferred into a pot containing orchid stone (50%) and wood bark (50%); acclimatized variegated (**W**), light-green (**X**), and dark-green (**Y**) plantlets. Bar: (**A**–**C**,**F**–**Y**) 1 cm; (**D**,**E**) 0.5 mm.

Currently, only two reports exist in the literature on the in vitro propagation of *C. lancifolium* [18,19]. The authors propagate *C. lancifolium* by culturing rhizomes obtained from asymbiotically germinated seeds on a semi-solid Hyponex medium containing 2 g/L peptone and 30 g/L sucrose. However, the percentage of seed germination has not been reported. Additionally, the in vitro propagation of variegated *C. lancifolium* has not been documented. Variegated *C. lancifolium* plants (Figure 1A,B) exhibit unique leaf variegation characterized by a mix of green, yellow, and white sectors distributed along the leaf blades. The variegation pattern is primarily striated, featuring longitudinal stripes that run parallel to the leaf veins. Some leaves present broader yellowish-white sectors, while others showcase finer green and yellow striping. Although *C. lancifolium* is known for its attractive foliage, the variegated form is particularly sought after due to its distinctive leaf patterns. The commercial production of variegated *C. lancifolium* through conventional propagation methods is challenging, including low propagation rates and extended time requirements. Therefore, this study aims to establish efficient in vitro propagation techniques for variegated *C. lancifolium* via asymbiotic seed germination. We have successfully developed in vitro propagation techniques for variegated *C. lancifolium* using asymbiotic seed germination for the first time. The present study not only provides a solution to traditional propagation challenges but also promotes the commercial cultivation of this orchid species. This protocol enables large-scale propagation, representing a significant advancement in orchid cultivation methods.

## 2. Results

### 2.1. Effects of PGRs and CW on Asymbiotic Seed Germination

The viability of variegated *C. lancifolium* seeds was 82%, but only 0.2% of the seeds germinated in MS medium without PGRs and CW. The addition of auxins (indole-3-acetic acid (IAA) or NAA) enhanced the seed germination rate. Variegated *C. lancifolium* seeds (Figure 1C,D) swelled within a month of cultivation (Figure 1E). The formation of white and green protocorms was observed after two months (Figure 1E,F), and the protocorms subsequently developed into green rhizomes (Figure 1G) after four months of cultivation. We also obtained many yellowish-green and a few white rhizomes. The percentage of seed germination increased in a dose-dependent manner following IAA supplementation. Increasing the concentration of IAA from 0.5 to 8.0 µM increased the germination rate from 0.7% to 10.2%. Similarly, NAA enhanced germination, reaching a maximum of 16.8% at 4.0 µM. However, increasing NAA concentration to 8.0 µM reduced the germination percentage to 12.6% (Table 1). Further enhancement occurred with the addition of CW to the medium containing NAA (4.0 µM). Among the tested CW concentrations (25–100 mL/L), 50 mL/L yielded the highest germination rate (26.2%), while both lower (25 mL/L) and higher (75 and 100 mL/L) concentrations were less effective. The combination of NAA (4.0 µM) and CW (50 mL/L) with kinetin (KN) further boosted germination, with the highest response (32.2%) recorded at 2.3 µM KN. Increasing KN concentrations beyond this level did not significantly enhance germination (Table 1). Notably, adding gibberellic acid (GA_3_ 0.3–2.9 µM) to the optimized NAA (4.0 µM) + KN (2.3 µM) + CW (50 mL/L) treatment significantly improved the germination rate. A progressive increase in germination occurred with increasing GA_3_ concentrations, with the highest percentage (46.8%) achieved at 2.9 µM GA_3_ (Table 1).

### 2.2. Effects of Auxins on Rhizome Proliferation

The rhizomes (dark green, yellowish-green, and white) obtained from the seed germination medium containing MS + NAA (4.0 µM) + KN (2.3 µM) + CW (50 mL/L) + GA_3_ (2.9 µM) were used as explants for rhizome proliferation. The rhizomes of variegated *C. lancifolium* (Figure 1H) developed branches within a month of cultivation (Figure 1F). After 6 months of cultivation on MS medium lacking auxins, the number (1.3) and length (0.5 cm) of the rhizomes were reduced. The addition of auxin enhanced rhizome growth and proliferation. Among the three auxins tested, NAA was the most effective, particularly at 5.0 µM, which yielded the highest number of rhizomes (17.4) with a corresponding length of 2.1 cm (Table 2, Figure 1J). This was followed by 10.0 µM NAA and 10.0 µM IBA, which also resulted in substantial rhizome multiplication (12.4 and 11.0, respectively), although with slightly shorter lengths (1.6 and 1.7 cm). The longest rhizomes were observed at 5.0 µM IBA (2.5 cm) and 2.5 µM NAA (2.4 cm), indicating that both IBA and NAA effectively promoted rhizome elongation and multiplication at specific concentrations. In contrast, IAA was the least effective auxin, producing relatively fewer and shorter rhizomes at all tested concentrations. The maximum response for IAA was recorded at 15.0 µM, resulting in 6.2 rhizomes with an average length of 1.1 cm. ANOVA results confirmed significant main effects of auxin type, concentration, and their interaction on both rhizome number (R^2^ = 0.748) and length (R^2^ = 0.491) (Table 2).

**Figure 2:**
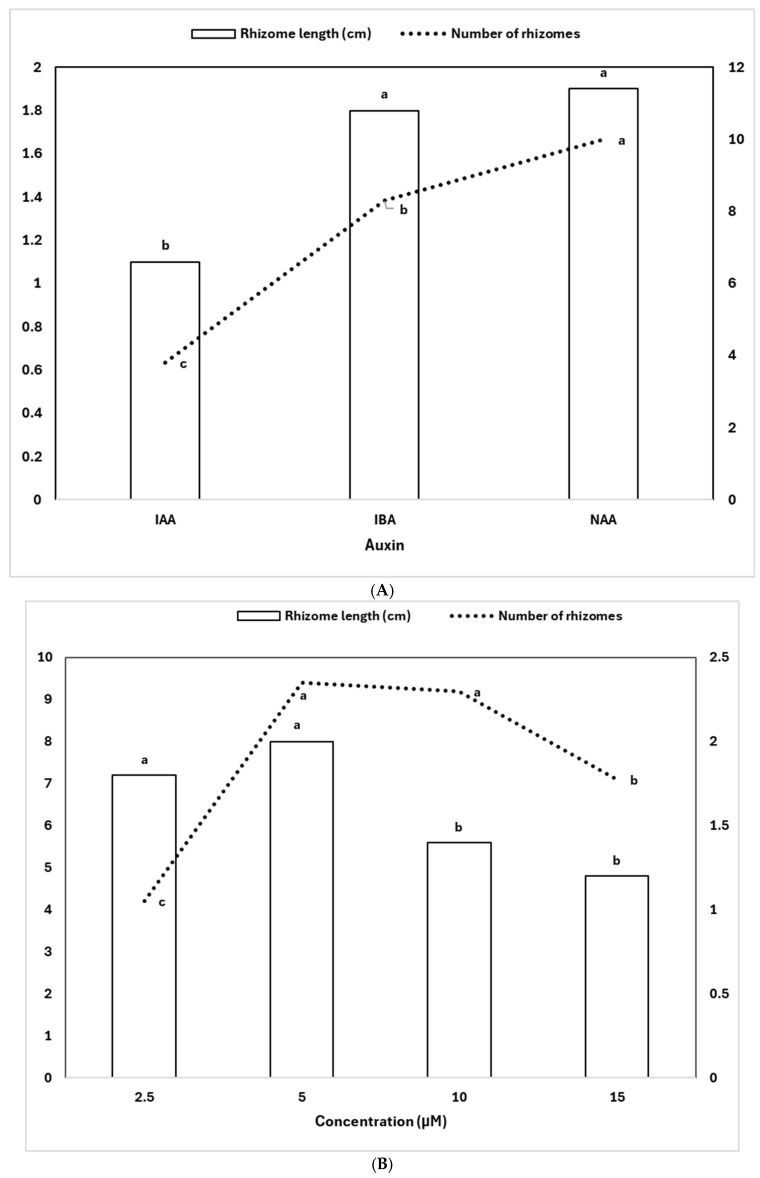
Effects of auxin type (**A**) and concentration (**B**) on rhizome growth and proliferation. Different letters indicate significant differences by DMRT at *p* < 0.5.

The type of auxin and Its concentration significantly Influenced both the number and length of rhizomes. Among the tested auxins, NAA produced the highest average number of rhizomes (10.0), followed by IBA (8.3), while IAA produced the lowest number (3.8). A similar trend was observed for rhizome length, with NAA (1.9 cm) and IBA (1.8 cm) promoting significantly greater elongation compared to IAA (1.1 cm) (Figure 2A). Regarding concentration, the highest rhizome number was recorded at 5.0 µM (9.4) and 10.0 µM (9.2), both significantly greater than 2.5 µM (4.2) and 15.0 µM (7.1). Rhizome length peaked at 5.0 µM (2.0 cm) and 2.5 µM (1.8 cm), while higher concentrations (10.0 and 15.0 µM) led to reduced elongation (Figure 2B). These results indicate that both auxin type and concentration interact to affect rhizome induction, with NAA at moderate concentrations (5.0–10.0 µM) being the most effective in promoting both rhizome number and length.

### 2.3. Effects of Cytokinins on Plantlet Regeneration

Rhizome segments cultured on MS medium without cytokinin were ineffective in inducing shoot buds. However, the addition of cytokinin promoted the regeneration of plantlets. The rhizome explants produced many rhizomes, and the growing tips developed shoot buds within four months, subsequently developing roots at the bottom of the shoots. The rhizome explants yielded 95% green plantlets, 4% light green plantlets, and 1% variegated plantlets (Figure 1K–R). Furthermore, the green plantlets (Figure 1N) grew faster than the light-green (Figure 1L,M,O) and variegated plantlets (Figure 1K,P,Q,R). The regeneration of plantlets was significantly affected by 2-IP, BA, and KN (Table 3). Among the tested cytokinins, N6-benzyladenine (BA) was the most effective in promoting plantlet regeneration. The highest regeneration percentage (79.4%) was observed at 10.0 µM BA, which also resulted in the greatest number of plantlets per rhizome (4.4) and significant plantlet height (8.5 cm). Increasing BA concentration from 2.5 to 10.0 µM enhanced regeneration and plantlet proliferation, while a further increase to 20.0 µM caused a decline in both parameters. KN at 5.0 µM also supported high regeneration (46.7%) and plantlet height (9.5 cm), although the number of plantlets per rhizome (2.2) was lower than that observed with BA. Similarly, N6-(2-isopentenyl)adenine (2-IP) induced moderate responses, with 5.0 µM yielding 38.9% regeneration and 2.1 plantlets per rhizome. However, at higher concentrations (10.0–20.0 µM), both 2-IP and KN showed reduced effectiveness (Table 3). Plantlet height varied depending on cytokinin type and concentration, with the tallest plantlets recorded in BA (10.2 cm at 5.0 µM) and KN (9.5 cm at 5.0 µM) treatment. Statistical analysis revealed highly significant effects of cytokinin type, concentration, and their interaction on all three parameters, with the highest R^2^ value observed for plantlet regeneration (0.919), indicating a strong treatment responsiveness (Table 3).

**Figure 3:**
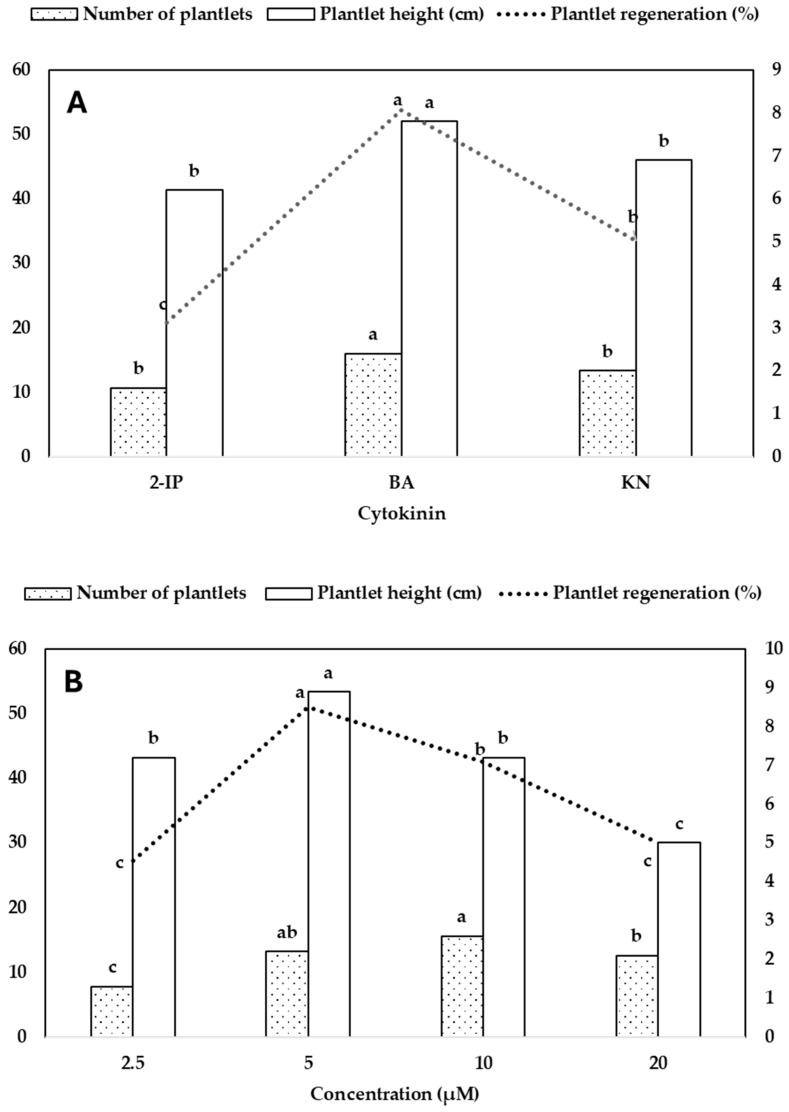
Effect of cytokinin type (**A**) and concentration (**B**) on plantlet regeneration (%), number of plantlets, and plantlet height (cm). Different letters indicate significant differences by DMRT at *p* < 0.5.

Cytokinin type and concentration significantly affected plantlet regeneration, the number of plantlets per rhizome, and plantlet height. Among the cytokinins tested, BA was the most effective, leading to the highest plantlet regeneration percentage (53.8%), the greatest number of plantlets (2.4), and the tallest plantlets (7.8 cm), followed by KN and 2-IP, which displayed moderate responses. KN promoted 33.5% regeneration, yielding 2.0 plantlets per culture and a mean height of 6.9 cm, while 2-IP was the least effective, showing 20.8% regeneration with shorter plantlets (6.2 cm) and fewer shoots (1.6) (Figure 3A). In terms of concentration, 5.0 µM cytokinin produced the most favorable results across all parameters, including the highest plantlet height (8.9 cm), high regeneration percentage (51.1%), and a significant number of plantlets (2.2). A concentration of 10.0 µM also supported a high number of plantlets (2.6), although with slightly lower regeneration (42.6%) and plantlet height (7.2 cm). Both lower (2.5 µM) and higher (20.0 µM) concentrations were less effective, particularly the 20.0 µM, where plantlet height significantly decreased (5.0 cm) (Figure 3B). These findings suggest that BA concentrations of 5.0–10.0 µM are optimal for plantlet regeneration and growth in rhizome-derived cultures.

### 2.4. Acclimatization of Plantlets

**Figure 4:**
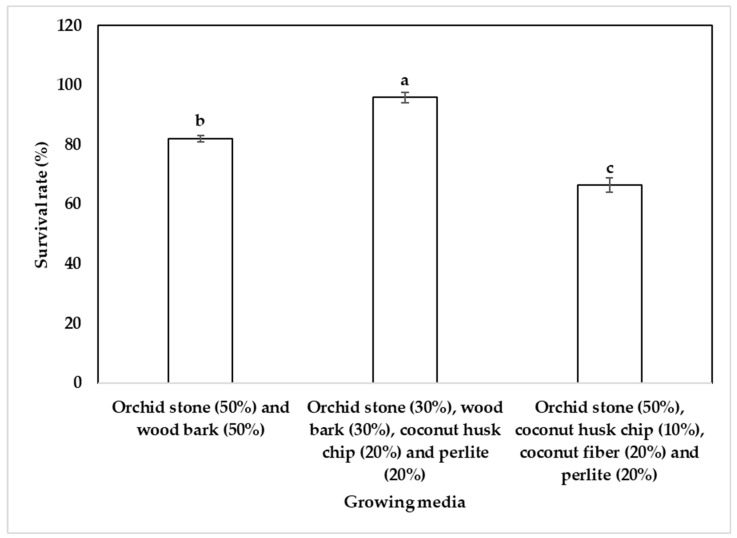
Effect of growing media on the survival rate of variegated *C. lancifolium* plantlets during acclimatization. Different letters indicate significant differences by DMRT at *p* < 0.5.

The survival rate of regenerated orchid plantlets during the acclimatization phase varied significantly depending on the potting substrate composition. The highest survival percentage (95.9%) was observed in a medium composed of orchid stone (30%), wood bark (30%), coconut husk chip (20%), and perlite (20%) (Figure 1S and Figure 4), indicating that a well-balanced mix of moisture retention and aeration components supports optimal plantlet establishment. A substrate consisting of orchid stone and wood bark in equal proportions (50:50) also resulted in high survival (82.1%) (Figure 1T), while a mixture of orchid stone (50%), coconut husk chip (10%), coconut fiber (20%), and perlite (20%) showed reduced survival (66.4%) (Figure 1U), possibly due to over-retention of moisture or inadequate aeration. After three months, the plantlets were transferred to pots containing orchid stone (50%) and wood bark (50%) (Figure 1V) because the plantlets grew best in this substrate mixture compared to the other two mixtures studied. These results underscore the importance of optimizing substrate composition to enhance the post-in vitro survival of orchid plantlets. The acclimatized variegated plantlets grew more slowly than the light green and dark green plantlets (Figure 1W–Y).

## 3. Discussion

Although orchid seed capsules contain many seeds, germination in nature is difficult because the seeds have undifferentiated embryos and lack the endosperm and fungal partner required for orchid seed germination [20]. This issue can be addressed using an in vitro seed germination technique, which allows orchid seeds to germinate asymbiotically in vitro, leading to successful plant regeneration [15,21]. However, asymbiotic seed germination of terrestrial *Cymbidium* species native to temperate regions is known to be challenging [22]. In this study, we demonstrate, for the first time, the in vitro propagation of variegated *C. lancifolium* through asymbiotic seed germination. The successful in vitro germination of this terrestrial orchid under asymbiotic conditions is influenced by PGRs and CW (Table 1). Auxin regulates germination and protocorm development in orchids during asymbiotic in vitro germination [23]. The inclusion of auxin, particularly NAA, promoted the germination of variegated *C. lancifolium* seeds in vitro. The beneficial effect of NAA on asymbiotic seed germination has been reported in various *Cymbidium* species, including *Cymbidium aloifolium* [24], *C. elegans* [12], and *C. nanutum* [17].

Various organic supplements, such as banana pulp, casein hydrolysate, CW, peptone, pineapple juice, potato homogenate, tomato juice, and yeast extract, are often added to the culture medium to enhance asymbiotic seed germination of orchids in vitro and subsequent growth and development [25,26]. In this study, incorporating CW into a medium containing auxin (NAA 4.0 µM) improved the germination of variegated *C. lancifolium* seeds in vitro. CW, known as liquid endosperm, promotes orchid seed germination due to its reported content of various growth-promoting substances, such as abscisic acid, amino acids, IAA, GA, minerals, proteins, sugars, vitamins, and zeatin riboside [25,27,28,29]. The beneficial effect of CW on asymbiotic seed germination has also been noted in *Cymbidium aloifolium* [30], *C. devonianum* [31], *C. findlaysonianum* [16], and *Cymbidium* hybrids [32]. However, the composition of coconut water, often used as a natural additive in in vitro culture media, can vary greatly depending on factors such as coconut maturity, variety, season, and environmental conditions. This variation makes it difficult to achieve consistent experimental results, even when coconut water is obtained from commercial suppliers intended for plant tissue culture. As a result, the lack of standardization can hinder the ability to replicate findings across different studies, complicating the interpretation of results. To address these issues, it is essential to include well-defined concentrations of nutrients, especially amino acids, vitamins, auxins, and cytokinins. This supplementation not only creates a more controlled environment for plant growth but also enhances the effectiveness of culture media, leading to more reliable and consistent outcomes in plant tissue culture experiments.

It has been reported that the supplementation of auxin (NAA) and cytokinin (BA/BAP) in the culture medium demonstrated a better response for asymbiotic seed germination of *Cymbidium aloifolium* [15,33], *C. faberi* [34], *C. devonianum* [31], *C. eburneum* [35], *C. elegans* [12], *C. iridioides* [13,36], and *C. lowianum* [37]. In contrast, a combination of BAP and 2,4-dichlorophenoxyacetic acid (2,4-D) decreased the percentage of seed germination in *Cymbidium giganteum* [11]. In this study, a combination of NAA (4.0 µM) and KN (2.3 µM) enhanced the germination of variegated *C. lancifolium* seeds. This result is in accordance with that of Kang and Yang [38], who reported that 14 *Cymbidium goeringii* F1 hybrid seeds germinated on a medium containing 0.1 mg/L NAA and 0.01 mg/L KN. GA_3_ is a gibberellic acid used to promote in vitro asymbiotic seed germination of orchids [39,40]; however, the effect of GA_3_ on seed germination in *Cymbidium* species has not been disclosed. In this study, variegated *C. lancifolium* seeds germinated best (46.8%) on a medium containing 50 mL CW, 4.0 µM NAA, 2.3 µM KN, and 2.9 GA_3_. Similarly, *Comparetia falcata* seeds achieved the highest germination rate (100%) on a medium with 15 µM of both KN and GA_3_ [41]. In another study, *Gastrochilus matsuran* seeds germinated best (93.3%) on a medium containing 5% CW, 1 µM NAA, and 1.5 µM GA_3_ [42].

The formation of rhizomes is a key step in the in vitro propagation of terrestrial orchids, particularly temperate *Cymbidium* species. Auxins, especially NAA and IBA, enhance rhizome proliferation and growth in variegated *C. lancifolium*, likely by promoting cell elongation of rhizomes. The highest proliferation of variegated *C. lancifolium* rhizomes (17.4 rhizomes with a mean length of 2.1 cm per explant) occurred on MS medium containing 5.0 µM NAA (Table 2). The beneficial effects of NAA on rhizome multiplication and growth have also been reported in other *Cymbidium* species [43]; however, the optimal concentration of NAA varies among species: *Cymbidium aloifolium* (27.0 µM) [44], *C. faberi* (5.4 µM) [45], *C. forrestii* (10.8 µM) [46], and *C. sinense* (5.4 µM) [47]. Additionally, rhizome proliferation and growth of *C. kanran* were observed to be best on medium containing 54.0 µM and 5.4 µM of NAA, respectively [48].

Regeneration of variegated *C. lancifolium* plantlets from rhizome explants occurred only when the medium was supplemented with cytokinin; however, the production of plantlets was influenced by the type and concentration of cytokinins. Among the cytokinins studied, BA at 10.0 µM was the most effective for plantlet regeneration. With this treatment, 79.4% of rhizome explants developed an average of 4.4 plantlets that reached a height of 8.5 cm (Table 3). The requirement of cytokinin (BA) for shoot or plantlet regeneration from rhizome tips has been documented in *Cymbidium aloifolium* [49], *C. faberi* [45], *C. forrestii* [46], *C. lancifolium* [18], and *C. sinense* [47]. The plantlets obtained in this study exhibited a range of striking and distinct variegation patterns, each reminiscent of the variegated *C. lancifolium* mother plant. Some plantlets displayed prominent marginal and interveinal variegation with creamy-yellow stripes along the leaf edges and between the veins, while the midrib remained green (Figure 1K), suggesting a moderately consistent chimeric expression with an ornamental appeal. Others featured a broad central yellow stripe along the midrib, flanked by green margins (Figure 1P), indicating potential genetic fixation and enhancing their value as uniform, ornamental cultivars. Additionally, certain plantlets exhibited both central and marginal variegation, characterized by vivid yellow coloration along the midrib and thinner yellow bands along the leaf edges (Figure 1Q), making them strong candidates for commercial propagation. Finally, some exhibited mild and irregular sectorial variegation (Figure 1R), suggesting partial expression or unstable chimerism, which may offer opportunities for further selection and stabilization through clonal propagation.

Acclimatization of plantlets is a crucial final step in micropropagation, and the survival rate of *Cymbidium* species after hardening on different substrates varies significantly based on the medium used. For instance, *Cymbidium aloifolium* plantlets demonstrated an impressive survival rate of 83% when acclimatized in a mixture of peat moss and small pieces of brick [49]. In contrast, Park et al. [2] reported a 97% survival rate for *Cymbidium goeringii* plantlets hardened on sphagnum moss. In another study, *Cymbidium eburneum* plantlets showed a 70% survival rate when hardened on a mixture of brick, charcoal, and decaying litter at a ratio of 1:1:1, along with a layer of moss [35]. In contrast, *Cymbidium findlaysonianum* plantlets displayed a survival rate of 70% when acclimatized to coconut peat [16]. Additionally, Wang et al. [37] found that *Cymbidium lowianum* plantlets achieved a 92% survival rate when hardened on moss. In the present study, among the three different substrate mixes tested, the highest survival rate (95.9%) of variegated *C. lancifolium* plantlets was obtained using a mixture of orchid stone (30%), wood bark (30%), coconut husk chips (20%), and perlite (20%). These findings highlight the significant impact of substrate choice on the survival of *Cymbidium* plantlets during acclimatization.

## 4. Materials and Methods

### 4.1. Plant Materials and Surface Disinfection

The five-year-old variegated *Cymbidium lancifolium* plants, grown in a shaded polyhouse, were self-pollinated on 19 November 2021 (Figure 1A), and the seed capsules were collected on 14 September 2022 (Figure 1B). The seed capsules were placed in a 500 mL glass beaker containing 5 mL of liquid detergent (Pril, Chongju, Republic of Korea) and kept under running tap water for 20 min. They were subsequently washed with distilled water and air-dried. The washed capsules were surface-disinfected in a laminar airflow cabinet by soaking them in 70% ethanol (95%) for 3 min, followed by treatment with 10% sodium hypochlorite (9%, Daejung, Daejeon, Republic of Korea) for 15 min. Each treatment was followed by washing twice and four times, respectively, with sterile distilled water. Finally, the seed capsules were dipped in ethanol (99.9%, Daejung, Daejeon, Republic of Korea) for 30 s and then flamed off.

### 4.2. Asymbiotic Seed Germination

The single rib of the surface-disinfected seed capsule was dissected longitudinally, and the seeds were placed in a 500 mL culture bottle containing 150 mL of MS medium with vitamins, supplemented with 500 mg/L AC, 30 g/L sucrose, and 7 g/L plant agar (control medium), along with various concentrations of PGRs and coconut water (Coco xim 100% Pure Coconut Water, Thanh Thanh Cong Factory, Ben Tre, Vietnam) (Table 1). The pH was adjusted to 5.5–5.6 using 1N potassium hydroxide or 1N hydrochloric acid before autoclaving for 23 min at 123 °C. GA_3_ was filter-sterilized and added to autoclaved medium. The AC, MS medium, PGRs, plant agar, and sucrose were sourced from Duchefa, Haarlem, The Netherlands. Nine culture bottles were used for each treatment, each containing 500 ± 80 seeds. The cultures were maintained at 23 ± 2 °C under an 8-h photoperiod using white light-emitting diodes (WLEDs) with a photosynthetic photon flux density (PPFD) of 6–10 µmol s^−1^ m^−2^. The percentage of seed germination was recorded after four months of culture. A tetrazolium test was conducted to assess the seed viability of variegated *C. lancifolium* [50] prior to asymbiotic in vitro seed culture. Seeds of variegated *C. lancifolium* were immersed in a 1.0% (*w*/*v*) 2,3,5-triphenyl tetrazolium chloride solution and kept in the dark at 30 °C. After 24 h, the seeds were washed with distilled water to remove excess stain and observed under an optical microscope.

### 4.3. Rhizome Proliferation

The rhizomes (0.1 mm long) obtained from the germinated seeds were cultured on MS medium containing 500 mg/L AC, 50 mL/L CW, 7 g/L plant agar, 30 g/L sucrose, and 0–15 µM auxins (Table 2) for rhizome proliferation. After 3 months of culture, the rhizomes were transferred to fresh medium. The cultures were maintained at 23 ± 2 °C under a 12-h photoperiod using WLEDs with a PPFD of 10–14 µmol s^−1^ m^−2^. Fifteen culture bottles were used for each treatment, with each bottle containing 13 rhizomes. The number and length of rhizomes were recorded after 6 months of culture.

### 4.4. Plantlet Regeneration

The rhizomes separated from the clusters grown on MS medium containing 5.0 µM NAA were cut into segments measuring 1.0–1.3 cm long and placed on MS medium supplemented with 500 mg/L AC, 50 mL/L CW, 7 g/L plant agar, 30 g/L sucrose, and 0–20 µM cytokinins to induce plantlets. The cultures were maintained at 23 ± 2 °C under a 12-h photoperiod, with WLEDs providing a PPFD of 30–34 µmol s^−1^ m^−2^. Fifteen culture bottles were used for each treatment, with each bottle containing 10 rhizomes. After 3 months of culture, the rhizomes were transferred to a fresh medium. The percentage of plantlet regeneration, along with the number and height of plantlets, was recorded after 6 months of culturing.

### 4.5. Acclimatization

The well-developed plantlets (4–11 cm tall) were transplanted into trays containing a mixture of orchid stone and wood bark (50:50), orchid stone, wood bark, coconut husk chip, and perlite (30:30:20:20), or orchid stone, coconut husk chip, coconut fiber, and perlite (50:10:20:20). These were fertigated with a ¼ MS basal salt solution at 2-day intervals and maintained in the polyhouse at 23 ± 2 °C under a 16-h photoperiod at a 200 µmol m^−2^ s^−1^ PPFD supplied by a high-pressure sodium lamp (LU400 W/PSL, GE lighting, Cleveland, OH, USA) and a relative humidity of 70–80%. After 2 weeks, the plants were fertigated with Hyponex (N-P-K; 20-20-20, Hyponex Japan Crop., Osaka, Japan) solution (2.0 g/L). Three trays were used for each treatment, with each tray containing 75 plantlets. The survival rate was recorded after 2 months. The acclimatized plantlets (3 months old) were transferred to plastic pots containing a mixture of orchid stone and wood bark (50:50) for further growth and development. They were maintained in a shaded polyhouse greenhouse and fertigated with a Hyponex solution.

### 4.6. Statistical Analysis

All experiments were conducted using a completely randomized design. The data were analyzed using ANOVA, and the means were separated using DMRT at the 5% level in SAS Release 9.4.

## 5. Conclusions

This study demonstrated that PGRs combined with CW, auxins, cytokinins, and substrates play crucial roles in in vitro seed germination, rhizome proliferation, plantlet regeneration, and plantlet survival. NAA, especially at 5.0–10.0 µM, was the most effective in stimulating rhizome multiplication in terms of both number and length. For plantlet regeneration from rhizomes, BA at 10.0 µM was optimal, promoting the highest regeneration percentage, plantlet number, and plantlet height. Among the tested substrates, a mixture of orchid stone (30%), wood bark (30%), coconut husk chips (20%), and perlite (20%) provided the highest acclimatization success (95.9%) and is recommended for ensuring the efficient transition of in vitro-regenerated plantlets to ex vitro conditions. These findings provide a reliable framework for developing an effective micropropagation protocol for variegated *C. lancifolium*. The stable and visually distinct variegation patterns observed among in vitro-derived plantlets from a variegated mother plant suggest the potential fixation of variegation traits during plantlet development. This indicates that variegation may have a genetic or chimeric basis rather than being solely epigenetic or stress-induced. However, molecular analysis is needed to confirm the genetic fidelity of variegated plantlets. The uniform expression of central, marginal, or interveinal variegation in individual plantlets enhances their ornamental value and provides a platform for selecting elite lines for commercial propagation. These findings also open new avenues for studying the mechanisms of variegation in orchids and their heritability through sexual and asexual propagation.

## Figures and Tables

**Table 1:** Effect of PGRs and CW on asymbiotic seed germination in variegated *C. lancifolium*.

PGRs (µM)				Coconut Water (mL/L)	Seed Germination (%)
IAA	NAA	KN	GA_3_		
0.0	0.0	0.0	0.0	0.0	0.2 ± 0.1 l
0.5	0.0	0.0	0.0	0.0	0.7 ± 0.3 l
1.0	0.0	0.0	0.0	0.0	2.2 ± 0.1 kl
2.0	0.0	0.0	0.0	0.0	5.4 ± 0.5 jk
4.0	0.0	0.0	0.0	0.0	8.3 ± 1.2 ij
8.0	0.0	0.0	0.0	0.0	10.2 ± 1.1 hi
0.0	0.5	0.0	0.0	0.0	3.0 ± 0.4 kl
0.0	1.0	0.0	0.0	0.0	6.2 ± 0.8 ijk
0.0	2.0	0.0	0.0	0.0	8.9 ± 1.1 hij
0.0	4.0	0.0	0.0	0.0	16.8 ± 1.5 f
0.0	8.0	0.0	0.0	0.0	12.6 ± 1.3 gh
0.0	4.0	0.0	0.0	25	18.1 ± 1.1 ef
0.0	4.0	0.0	0.0	50	26.2 ± 2.0 d
0.0	4.0	0.0	0.0	75	20.9 ± 1.6 e
0.0	4.0	0.0	0.0	100	14.3 ± 1.3 fg
0.0	4.0	2.3	0.0	50	32.2 ± 1.7 c
0.0	4.0	4.7	0.0	50	27.8 ± 1.5 d
0.0	4.0	9.4	0.0	50	28.3 ± 1.7 d
0.0	4.0	2.3	0.3	50	33.3 ± 2.0 c
0.0	4.0	2.3	1.4	50	40.6 ± 1.8 b
0.0	4.0	2.3	2.9	50	46.8 ± 1.8 a

Means ± SE followed by different letters (a–l) are significantly different according to Duncan’s multiple range test (DMRT) at *p* < 0.5.

**Table 2:** Effect of auxins on growth and proliferation of rhizomes of *C. lancifolium*.

Auxin (µM)			Number of Rhizomes	Rhizome Length (cm)
IAA	IBA	NAA		
0.0	0.0	0.0	1.3 ± 0.2 h	0.5 ± 0.1 f
2.5	0.0	0.0	1.9 ± 0.4 gh	1.0 ± 0.1 ef
5.0	0.0	0.0	2.8 ± 0.4 gh	1.4 ± 0.2 cde
10.0	0.0	0.0	4.1 ± 0.6 fg	1.0 ± 0.1 ef
15.0	0.0	0.0	6.2 ± 1.0 ef	1.1 ± 0.2 de
0.0	2.5	0.0	3.0 ± 0.6 gh	2.0 ± 0.2 ab
0.0	5.0	0.0	8.0 ± 1.0 de	2.5 ± 0.1 a
0.0	10.0	0.0	11.0 ± 1.0 bc	1.7 ± 0.2 bc
0.0	15.0	0.0	5.8 ± 0.7 ef	1.2 ± 0.2 cde
0.0	0.0	2.5	7.8 ± 0.8 de	2.4 ± 0.2 a
0.0	0.0	5.0	17.4 ± 0.9 a	2.1 ± 0.2 ab
0.0	0.0	10.0	12.4 ± 1.0 b	1.6 ± 0.2 bcd
0.0	0.0	15.0	9.4 ± 0.9 cd	1.3 ± 0.2 cde
ANOVA	R-Square		0.748	0.491
	Coefficient of variation		34.86	32.94
	Auxin		*p* < 0.0001	*p* < 0.0001
	Concentration		*p* < 0.0001	*p* < 0.0001
	Auxin * Concentration		*p* < 0.0001	*p* < 0.0208

Means ± SE followed by different letters (a–h) are significantly different according to DMRT at *p* < 0.5. * is interaction sign.

**Table 3:** Effect of cytokinins on inducing plantlets from *C. lancifolium* rhizomes obtained through asymbiotic seed germination.

Cytokinin (µM)			Plantlet Regeneration (%)	Number of Plantlets per Rhizome Culture	Plantlet Height (cm)
2-IP	BA	KN			
0.0	0.0	0.0	0.0 ± 0.0 j	0.0 ± 0.0 g	0.0 ± 0.0 i
2.5	0.0	0.0	21.7 ± 2.0 fg	1.1 ± 0.1 f	5.9 ± 0.6 e–h
5.0	0.0	0.0	38.9 ± 1.6 e	2.1 ± 0.3 b–e	7.0 ± 0.4 c–e
10.0	0.0	0.0	13.9 ± 1.6 hi	1.7 ± 0.2 c–f	6.3 ± 0.6 e–g
20.0	0.0	0.0	8.9 ± 1.4 i	1.6 ± 0.2 c–f	5.5 ± 0.5 f–h
0.0	2.5	0.0	33.9 ± 3.2 e	1.3 ± 0.2 ef	7.6 ± 0.5 cde
0.0	5.0	0.0	67.8 ± 3.0 b	2.3 ± 0.2 bc	10.2 ± 0.9 a
0.0	10.0	0.0	79.4 ± 1.8 a	4.4 ± 0.5 a	8.5 ± 0.5 a–c
0.0	20.0	0.0	61.7 ± 2.0 c	2.7 ± 0.3 b	4.9 ± 0.5 gh
0.0	0.0	2.5	26.1 ± 2.3 f	1.4 ± 0.2 def	7.9 ± 0.7 bcd
0.0	0.0	5.0	46.7 ± 2.4 d	2.2 ± 0.4 bcd	9.5 ± 0.8 ab
0.0	0.0	10.0	34.4 ± 1.5 e	1.8 ± 0.3 c–f	6.7 ± 0.5 d–g
0.0	0.0	20.0	19.4 ± 2.1 gh	2.0 ± 0.3 b–e	4.4 ± 0.3 h
ANOVA	R-Square		0.919	0.537	0.503
	Coefficient of variation		17.62	40.01	25.37
	Cytokinin		*p* < 0.0001	*p* < 0.0001	*p* < 0.0003
	Concentration		*p* < 0.0001	*p* < 0.0001	*p* < 0.0001
	Cytokinin * Concentration		*p* < 0.0001	*p* < 0.0001	*p* < 0.0171

Means ± SE followed by different letters (a–j) are significantly different according to DMRT at *p* < 0.5. * is interaction sign.

## Data Availability

Data is available within the article.

## Abbreviations

The following abbreviations are used in this manuscript:

AC	Activated charcoal
CW	Coconut water
GA_3_	Gibberellic acid
IAA	Indole-3-acetic acid
IBA	Indole-3-butyric acid
KN	Kinetin
MS	Murashige and Skoog
2-IP	N^6^-(2-isopentenyl)adenine
BA	N^6^-benzyladenine
PPFD	Photosynthetic photon flux density
PGRs	Plant growth regulators
WLEDs	White light-emitting diodes
NAA	α-naphthalene acetic acid
